# On the Density of Enamel as an Element in Determining the Proper Time to Devitalize the Tooth Pulp

**Published:** 1883-08

**Authors:** J. Edw. Bine

**Affiliations:** Rochester, N. Y.


ARTICLE III.

### ON THE DENSITY OF ENAMEL AS AN ELEMENT IN DETERMINING THE PROPER TIME TO DEVITALIZE THE TOOTH-PULP.

BY J. EDW. BINE, D. D. S., ROCHESTER, N. Y.

Late in '79 a little Miss of fourteen years was brought to us to have her teeth examined, and, if necessary, put in repair. She was exceedingly delicate in her general make-up, and to that degree that we immediately came to the conclusion that a corresponding delicacy would show itself in the teeth. And so it proved. The arches were handsomely curved, the upper and lower sets perfectly articulated, the individual teeth finely outlined, the color what might be appropriately termed "milk and water," the texture such as would readily give way to cutting instruments of whatever kind. We regarded this case as one in which filling and re-filling would necessarily be the treatment for some years to come, provided decay had up to this time made any progress. The examination brought to light several cavities in the incisors, and as many more in the molars. We decided, of course, to fill. But with what? The mother objected to gold because of its color, but would have that material used if in our judgment it was thought best. This objection was regarded, from a practical point of view, as of little or no consequence, and was so stated; nevertheless it was not thought best to fill with gold, and for the following considerations: First, the certainty of failure with any material to save these teeth from the recurrence of decay; second, the belief on our part that the preparation of the cavities would entail a greater amount of physical and mental worry than the little patient could be expected to withstand; third, that it would call for the payment and repayment of a comparatively high fee, whereby might be slain the bird that lays the greenback egg; fourth the total lack of that self-sacrificing spirit that is ever willing to shoulder the blame that usually attaches to the dentist when fillings fail, no matter whether the fault lie in defective manipulation, or in defects inherent in the material operated upon. In view of these considerations we decided to fill the front teeth with gutta-percha, and the others with tin-foil. We filled. We at the same time reminded both parent and child that re-filling might become necessary, possibly in a short time; and urged frequent visits to the office that anything wrong might be righted in good season.

According to expectation, hardly six months had passed when the case presented for the re-filling of one of the front teeth. The margins of the cavity had crumbled, and the gutta-percha had given out. When about to examine the cavity, our attention was arrested by the color of the teeth, which was that of a set somewhat of a stranger to tooth-brush and powder. In answer to our remark that since her previous visit she had evidently given her teeth very little attention, she said that she had brushed them as regularly and as carefully as ever, and notwithstanding their yellowish appearance they had not become that color through neglect. This statement we very soon verified, for we found that the changed appearance was due not to anything *on* the teeth, but to something *in* them. It would not rub off, and was not to be got out without the tooth. Here was a case in which six month's time had brought about a change from delicate "milk-and-water" teeth to teeth whose yellowish color and firm texture placed them among the strong and durable. Without a doubt the enamel had increased in density, which condition was easily accounted for by the generally healthful look of the child and her changed manner—formerly listless and destitute of energy, now interested in everything and full of vitality.

Now it so happened that at this time we had our hands full of pulpless teeth, teeth with pulps dead, pulps dying, and pulps that promised anything but good behavior, out of alcohol. Every case—so it seemed at least—that presented at this time was for pulp treatment of some kind. Evidently pulps were " on a tear." The thought occurred to us—Suppose we had destroyed the pulp of one of these incisors when their treatment first fell to us, and suppose that instead of growing denser the enamel had become less dense; or suppose we were to destroy a pulp now in this case, and the next six months bring about a change the opposite of that of the six months past, what would be the result?

Every one in active practice has met with cases to which the following descriptions will apply: A man of fifty years calls to have a crown set on an incisor root. We see at a glance that the other teeth are good, and readily infer that something out of the usual order has happened in the case of this particular tooth. In answer to our inquiries as to the cause of its demoralized state, he says that when a lad of fifteen he was struck with a stone, that he bit a pin, or that a traveling dentist discovered a black speck in or on the enamel near the gum, drilled a hole in the part and filled it, and the tooth soon grew darker than the others, and finally crumbled to its present crownless condition. Our conclusion is that previous to the change of color in the tooth, the pulp had died, and propably from the cause named.

Another man of fifty years calls. He wants a full set of artificial teeth. We examine his mouth and find one upper incisor tooth, discolored, ragged, and worse as to looks than none at all. In accounting for the discoloration he names the same cause as the aged party examined a little before; and though this is a badly damaged tooth, he unnecessarily assures us that it has outlived all of its neighbors, every one of which gradually melted away " without apparent cause or provocation," and their remains were removed from time to time by the dentist.

Here we have two men of fifty, one of them the possessor of thirty-one good teeth and one badly decayed root; the other, of one solitary tooth and that not of the best. Both men met with like accidents at like ages, and are now of the same age. Between these times they have lived, moved, and had a being, each like the other in every important particular. Then why this difference as to their dental possessions? The reason must be looked for in conditions that prevailed at the time of the accident. The man with the thirty-one teeth and one root was in poor condition. Nutrition, because of the quality or quantity of material, or the systems inability to appropriate such material, was at ebb tide—going, but not necessarily quite gone. And while the death of the pulp cut off for good the main supply of nutritive material in the unfortunate tooth, the nutrition of the other teeth was merely suspended. On the return of general health the teeth with pulps increased in density, and that density was maintained, with the help of alkaline or neutral oral secretions, or possibly in spite of such secretions, if acid.

The man with the solitary tooth was in good condition at the time of the accident; nutrition was, and had been at its best. In his case it was flood tide, with a possible further rise; and though, as in the case first cited, the accident deprived the tooth of all means of nutritive supply, except through the peridental membrane, the tooth had locked up within itself that on which it could feed till fifty. But it may be asked why this tooth, with its principal means of nutrition cut off, should outlive its associates whose pulps had escaped injury until very late in life? The answer is —Up to the time of the accident all the teeth were supplied by the same means and from the same sources; but after the accident, while the pulpless tooth was compelled to draw its nutritive supply wholly through the peridental membrane, it could lose what it thus gained and what it had already stored up only through the same organ. Change for the worse in the general health interferred with the nutrition of all, and there was waste of material already stored up in the teeth, but not in the same degree. While all were losing some of their salts through the solvent action of the vitiated oral secretions, those with pulps were losing more through the activity of the absorbent elements of these organs.

A glance at the minute anatomy of a tooth, and a little thought as to some of the functions of the several parts, may make this more clear. In the root we have the pulp in communication with the peridental membrane through and by no means of the soft fibre of Tomes, the contests of the inter-globular spaces, and the lacunæ. In the crown we find it in communication with the outer surface of the enamel through and by means of the soft fibre of Tomes, the contents of the inter-globular spaces, and the less than three per cent. of organic matter constituting the lace-like matrix in, around, by and through which the salts of enamel are deposited. Now all deposits of new material must be through the pulp or peridental membrane. All solvent effects may be through the oral secretions, or they may be through such secretions and the pulp and peridental membrane. Where well-made teeth are acted upon by the secretions only, their solution is comparatively slow; but where subjected to the action of such secretions in conjunction with the nutritive inactivity or the absorbent functions of the pulp, their term of usefulness is limited indeed.

If these things are true, it follows that the proper time to destroy the tooth-pulp—granting that it must be destroyed, and we have not the too frequent privilege of naming the time—is when the enamel is at its maxium density; for when it is not in in this condition the tooth may be safely regarded as comparatively short-lived, and particularly so in case of failure in general health and consequent suspen sion or retrogression in the nutritive function.—*Independent Practitioner*.

---

ARIICLE IV.

### EXPOSED PULP AND TREATMENT.

